# Detection and Classification System for Rail Surface Defects Based on Eddy Current

**DOI:** 10.3390/s21237937

**Published:** 2021-11-28

**Authors:** Tiago A. Alvarenga, Alexandre L. Carvalho, Leonardo M. Honorio, Augusto S. Cerqueira, Luciano M. A. Filho, Rafael A. Nobrega

**Affiliations:** 1 Electrical Engineering Department, Federal University of Juiz de Fora, Juiz de Fora 36036-900, Brazil; augusto.santiago@ufjf.edu.br (A.S.C.); luciano.ma.filho@gmail.com (L.M.A.F.); rafael.nobrega@ufjf.edu.br (R.A.N.); 2 MRS Logística, Juiz de Fora 36060-010, Brazil; alcarvalhox@gmail.com

**Keywords:** rail surface defects, eddy current, railway maintenance, rail grinding, wavelets, convolutional neural network

## Abstract

The prospect of growth of a railway system impacts both the network size and its occupation. Due to the overloaded infrastructure, it is necessary to increase reliability by adopting fast maintenance services to reach economic and security conditions. In this context, one major problem is the excessive friction caused by the wheels. This contingency may cause ruptures with severe consequences. While eddy’s current approaches are adequate to detect superficial damages in metal structures, there are still open challenges concerning automatic identification of rail defects. Herein, we propose an embedded system for online detection and location of rails defects based on eddy current. Moreover, we propose a new method to interpret eddy current signals by analyzing their wavelet transforms through a convolutional neural network. With this approach, the embedded system locates and classifies different types of anomalies, enabling an optimization of the railway maintenance plan. Field tests were performed, in which the rail anomalies were grouped in three classes: squids, weld and joints. The results showed a classification efficiency of ~98%, surpassing the most commonly used methods found in the literature.

## 1. Introduction

The railway is an essential and advantageous means of transport well consolidated in several countries. They act in the supply of cargo and goods for various commercial and production sectors and people’s transport. Nowadays, the degree of reliability in cargo transportation is ever-increasing due to personal safety, preventing loss of assets, meeting deadlines and reducing maintenance expenses. Furthermore, these factors have a direct impact on the market competitiveness.

The expansion of the number of loads and the increase of the rails’ use causes acceleration of the rails’ wear. This results in increased surface defects on the rails, altering the balance of contact between the rail and the wheels and consequently cause accidents and even derailment [1]. Accidents and severe damage can occur if the faults on the tracks are not repaired. Therefore, the detection and maintenance of railway defects are paramount issues in the operation of any railway system. Among all different areas, the literature shows an increasing number of works [1,2,3,4] that seek to improve maintenance stages by assigning reliability and logistics. With the development of research and new tools to assist in the railway system’s logistics and operation stages, the corrective maintenance steps have been replacing by preventive maintenance steps.

The rails are responsible for sustaining large loads, which results in structural wear. The defects can be worn-out rails, joint and weld problems, internal structural defects, corrugations and rolling contact fatigue (RCF) [1,5]. The RCF have various intensities, such as surface cracks, head checks, squats, spalling and shellings. Head checks are small thin cracks with a few millimeters; spalling or flacking is a degradation of the running part of the rail, usually associated with areas of high contact stress; and shelling occurs when parts of the gauge or the top rail are broken.

These types of defects are usually repaired through the rail grinding process, in some cases, by welding or replacing severely damaged sections [1,5]. Rail grinding is the method of roughing the rail using a machine with a certain number of grinding wheels, which removes the rails’ defects by removing metal from the surface. It is applied to restore the profile and remove irregularities from worn rails, prolonging its lifespan [5]. The critical point in the maintenance process is the determination of the ideal moment to perform the interventions, intensity and sections of the track.

The previous knowledge of the regions where the rails are worn and the type of wear that it possesses confers a significant advance in the maintenance process’s performance. This work presents a new method for detection and classification of the superficial defects of the rails. The method proposes an Eddy Current (EC) and wavelet-based tool to provide information on maintenance process planning and guidelines for the grinding stage.

Traditionally, the detection and classification of such problems are done manually. The specialized operators traverse the tracks seeking to visually identify the points with defects and classify the occurrences according to their experience. This realization leads to high costs and excessive time demand and does not produce a well-structured diagnostic. A mapping of the rails with the identification of the positions and more detailed faults provides a very relevant improvement to the maintenance process.

In the literature, there are some proposals that seek to assist in this activity. Different sensors are applied to track fault detection, such as EC-based, ultrasound, image processing and more. Moreover, different processing techniques, type of defect detected and classified intensity of failures, besides the application’s purpose can be found in the literature.

The work in [6] presents a system to detect squats on rails based on a device that monitors the train wheel’s oscillation frequency. In it, the sensor should be installed directly on the service trains. It uses wavelet power Spectrum in Frequencies between 1060 and 1160 Hz and a simple threshold for squat identification. This work is limited to squats detection for preventive maintenance.

Image processing is also applied in the field, as shown in [7]. This work presents an intelligent vision detection system, seeking image enhancement through nonlinear processing that improves illumination of the capture scene. Other applications based on track image analysis to identify surface defects can be seen in [8,9,10]. In [11], it is proposed a method to surface reconstruction of the rail from a 3D laser. It compares the reading profile with a pattern, and the points with large deviations are indicated as possible defects.

In [12], the authors present a method for detecting specific defects in welds to eliminate the hidden risk of breakage, making it possible to evaluate the welding step through parasitic current use. The presence of a defect is detected by applying a threshold in the evaluated signal. In this study, four types of welds anomalies are defined beyond the standard sample. The technique applied in detection and classification is the combination of Continuous Wavelet Transform (CWT) and Convolutional Neural Network (CNN). Two steps of this technique are used to detect the presence of the anomaly and perform its classification. This report does not detail how the sampling process was performed, and its application is aimed at welding analysis only.

In [13], the author used signals from accelerometers installed on the train to capture irregularities in the tracks, particularly in this case, 34 squat samples were collected and later analyzed using the wavelet transform. This method allows the identification and classification of this type of RCF with a prediction accuracy of 88.2%. The author did not detail the algorithm used, but it is possible to notice the application of prediction methods through wavelet transform in RCF signals.

In [14], the authors use a 16-channel matrix eddy current inspection device to inspect head check and shelling defects. Samples with known width and depth were used to calibrate the probes and elucidate the depth through the correlation with the phase of the eddy current signal obtained by the probes divided into real and imaginary parts. After equalizing the correlation, the 2D signal was compared to 3D signals, and an accuracy of + or −1 mm was proven. In this article, the author does not mention details of the algorithm used in the comparison but shows the potential of using eddy current as a potential solution for problems of this nature.

As mentioned above, this work presents a new embedded system capable of detection, location, and classification of rail surface anomalies along the track. It makes use of an EC as the base sensor, nonlinear filtering for online event detection, and CWT and CNN to perform the classification. The main novelties of this work are the embedded system for online detection and location of rails defects, and the automatic classification of the EC acquired signals based on CNN combined with Wavelet Power Spectrum (WPS). Although the application of metal surface wear detection through eddy current probes is widely used as a non-destructive method, the proposed embedded system is capable of automatic detection, location, and classification of rail surface anomalies, going a step further in preventive rail maintenance. Moreover, while recent works propose the use of wavelet transform combined with deep neural networks to process images of the rail surface, the proposed work relies on the EC acquired signal to keep the capability to detect and classify anomalies that are not visible.

The rest of this paper is organized as follows. In Section 2, the basic concepts of the types of rail defects and the maintenance procedures are presented. Section 3 presents the proposed method for detection and classification of rail surface defects. The implemented system and the field test performed to validate the proposed method are presented in Section 4. In Section 5, a discussion about the field test result is presented. The conclusions of this paper are given in Section 6.

## 2. Rail Surface Defects

The effective maintenance of a railway line’s components provides safety and eliminates wear and imperfections that can lead to accidents [1]. Railway operation requires efficient planning for maintenance. Process optimization for assessing the operational conditions of rails and locomotives is vital. In this way, all the tools that help evaluate rail’s defects and rolling stock are fundamental. Therefore, any technique or procedure that enables the identification and mapping of defects provides advances for railway safety and operation.

The accidents have been decreasing sharply due to the railway structure components practical inspection and maintenance components such as rail lubrication and rectification [15]. The work in [15] presents a general analysis regarding defects and their consequences on the railway. It shows that the costs caused by railway problems and accidents have decreased consistently due to the increased inspection process. Reducing costs associated with the inspection and maintenance processes is a challenge for the operation of the railway system. It can be achieved by improving tools and developing technologies that improve process performance. Another piece of essential information is provided in [15], which describes tooling in the structuring of problems currently requiring more resources and time. He points out that RCF problems are the primary focus on the maintenance process due to the increasing demand for railway work, which results in increased speed, loads and traffic density.

Squats is the name of RCF ’s defects starter on the rail surface. It is caused by a combination of high normal and tangential stresses between the wheel and the rail, which cause strong shear of the surface layer of the rail and fatigue or exhaustion of the material’s ductility [15].

**Figure 1:**
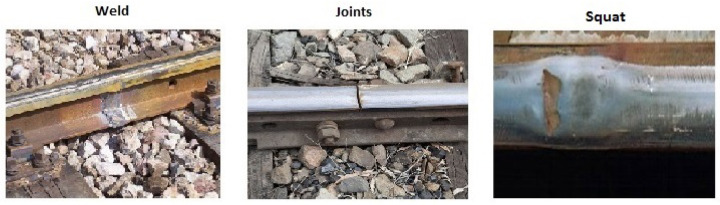
Types of anomalies considered.

In this work, three structural classes are considered to be detected and classified by the proposed method. Figure 1 shows the squat type defects along with welds and joint joints between rails. The latter two are not superficial defects but may generate false alarms in the detection process. Therefore, they were included as identification patterns enabling the respective characteristic. Besides, the mapping of points containing welds and joints allows the track’s maintenance to be improved.

Some characteristics of each of the classes evaluated by the method are given below to allow their identification.

### 2.1. Squat

It appears appear predominantly occurs in flat areas of the rail surface [1]. This crack grows progressively, branching horizontally below the bearing surface, separating it from the rail body. They are categorized according to the severity of the defect as follows: mild multiple gauge corner, moderate/medium running, severe/large running surface, severe and very severe/large multiple running surfaces.

### 2.2. Weld

Welding produces a weak point susceptible to irregularities, and it is present in the construction stage and the maintenance of rails. In the assembly of a new line, the joining of some rails is done by welding. Now, the maintenance step uses this process when replacing the railings.

### 2.3. Joint

Joints can be insulated or dried in order to avoid fractures due to rail expansion. They can perform electrical isolation between successive sections of the rail called the track circuit (TC) [16]. TC scans are used in the train detection process and in signaling control. Therefore, the mapping of these structures is of great importance for the track’s maintenance and management.

### 2.4. Maintenance—Rail Grinding

As stated in [5], grinding consists of intentional wear on the maintenance teams’ tracks. This practice aims to prevent the progress of surface wear caused by the rail wheel contact. They are divided into three instances: corrective, preventive and cyclic.

The grinding process is vital and guarantees good contact between the tracks and wheels. It is a slow procedure and requires the interruption of traffic requiring many hours of work and often excessive wear of the ways. Therefore, the mapping carried out by identifying the sections with defects adds the characterization of the wear intensity, thus allowing a gain of performance in the rail grinding process. Targeting through defect mapping would result in lower losses, higher productivity and longer rail life.

## 3. Proposed Method for Detection, Location and Classification of Rail Surface Defects

The proposed system aims to detect, classify and map the defects on the track’s surface. It brings structural information from the railway surface line that allows workers to carry out maintenance processes in the most efficient way. Therefore, it allows the elaboration of a schedule to perform the repair operations, evaluating the best way of acting is possible.

**Figure 2:**
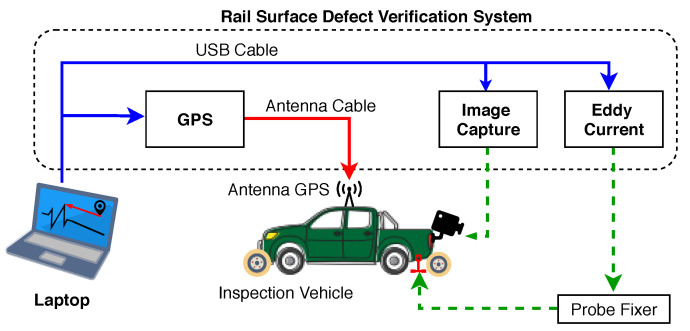
Rail surface defect verification system diagram.

Figure 2 shows a diagram with the integration of the Rail Surface Defect Verification System (RSDVS) components into the inspection vehicle. In it are installed the mechanical structure to fix the probes that perform the reading of the surface of the rail, a computer for the control and processing of data, a Global Positioning System (GPS) with positioning system for evaluating the positioning to capture the coordinates of the defects and a camera system to make acquisitions of the tracks and allow to check the detected defects.

**Figure 3:**
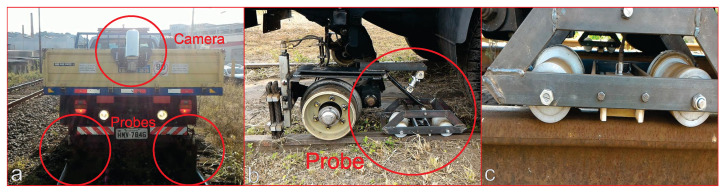
Probes installed in the inspection vehicle. (**a**) The camera and the two probes installed. (**b**) The probe attachment trolley. (**c**) A detailed view of the trolley.

Figure 3 shows details of the proposed system, where the inspection vehicle is equipped with RSDVS. On the left, it can be seen the camera installed on the top and two probes that perform the analysis through the EC. In the middle, it is possible to observe the probe attachment trolley. A detailed view of the attachment trolley can be seen on the right. These EC probes are fixed at a distance of ~2 mm from the rail surface.

**Figure 4:**
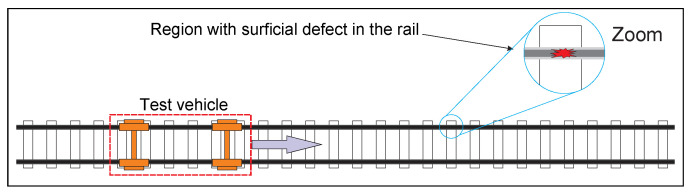
Vehicle track monitoring process illustration.

Figure 4 illustrates the process in which a railroad vehicle travels along the track searching for defects. The proposed system identifies the rail defect, informs the operator, stores the data and its geographic coordinates and acquires an image from the rails.

### 3.1. Rail Surface Defect Verification System

The proposed system aims to map the track’s defects through the identification of the regions with squats. Moreover, the knowledge of the points containing welds and joints on the railway line allows distinguishing between squats, adding important information for the track management and maintenance. To make this possible, the system must periodically monitor long stretches of the track.

On the railway, maintenance vehicles are widely used in various types of applications. Therefore, the proposed system was designed to be embedded into such vehicles.

The proposed system stores the signal from the EC probes, the vehicle route, the geographic coordinates and images of the detected rail defects.

**Figure 5:**
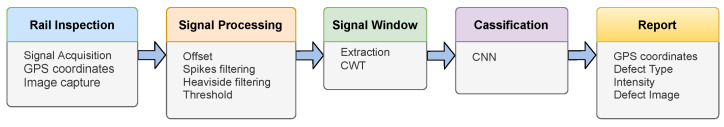
Step diagram for detecting surface defects on rails.

Figure 5 shows a diagram illustrating the steps taken to analyze the rail’s surface and map the faulty points. In general, the vehicle travels the section under analysis with constant speed between 5 and 30 km/h. The recommendation is the use of lower speed to decrease noise due to trepidation. Initially, the signal undergoes an adjustment to remove the baseline fluctuation. Nonlinear filtering is applied to remove noise and a threshold is applied to detect and locate the rail defects. The GPS stores the route information during the inspection and the coordinates of the located fault. These steps take place on-line during the rail inspection. Later, the classification of the type of surface defect is performed offline. In this step, windows of the filtered signal with the detected defects are selected. After that, the CWT is applied to the selected window. Finally, the CWT image feeds a CNN for rails defect classification.

After the inspection, a report is generated, which will be used later for rail maintenance, knowing the location and type of defect. In this way, it is possible to determine the amount of rail that should be roughed, acting more precisely.

The EC signal is acquired with a sampling rate of 7.5 kHz, where the frequency of the injected sinusoidal signal is 50 kHz. The EC equipment used allows generating signals in the frequency range from 20 Hz to 20 MHz. The GPS has an acquisition rate of 5 Hz, which is sufficient to map the entire trajectory.

### 3.2. Continuous Wavelet Transform

Spectral analysis of a stationary signal can be performed by Fourier transform (FT) [17]. The frequency content of these signals are not time-dependent. Conversely, the CWT produces a high-resolution time-frequency analysis. Therefore, the CWT can be used to analyze the non-stationary signal.

The CWT is a time-frequency transform, where the evaluated function is multiplied by a set of shifting and scaling functions [18,19]. It is a linear integral transform that can be used to explore non-stationary signals characteristics, being useful to extract information of variations in specific frequency bands, and to detect local structures. For a given signal, its integral transform is defined as

(1) $$w_{f}^{\psi }\left(a,\tau \right)=\frac{1}{\sqrt{a}}\int_{-\infty }^{\infty }f\left(t\right)\psi *\left(\frac{t-\tau }{a}\right)dt,$$

where $w_{f}^{\psi }\left(a,\tau \right)$ are the wavelet coefficients; *a* is a scaling factor, (a>0); f(t) is the analyzed signal; ∗ indicates a complex conjugate; ψ(t) a mother wavelet; and τ is a continuous variable and *t* sampling time of the analyzed signal.

The FT characterizes a signal from a series of sine waves with distinct frequencies. The CWT uses wavelet functions, which are small signals with different scales and located in time. The wavelet function and CWT are usually complex, having a real and imaginary part that can be represented as magnitude and phase [20]. The WPS allows you to describe the intensity of the energy contained in each frequency as shown:

(2) $$WPS={\left|W_{x}^{\psi }\left(a,\tau \right)\right|}^{2}.$$

where WPS is power spectrum of wavelet transform.

Equation (3) presents the complex Morlet wavelet that was used in this work. Therefore, applying the CWT method is informed as inputs the signal to be evaluated, mother wavelet $\psi \left(t\right)$ and the range of scales. A vector containing 511 elements starting at 1 with a unit step up to 512 was employed as the scale applied in the wavelet.

(3) $$\psi \left(t\right)=\frac{1}{\sqrt{\pi B}}\exp^{-\left(\frac{t^{2}}{B}\right)}\exp^{\left(2j\pi Ct\right)},$$

where *B* is the bandwidth and *C* is the center frequency.

### 3.3. Convolutional Neural Network

A convolutional neural network is a select type of multilayer neural networks [21]. It has high capacity to recognize existing patterns in images, where little computational effort is required to treat pixels. It is robust and can recognize patterns with significant variability, even with distortions and geometric transformations [22]. Therefore, it configures an excellent tool to characterize the patterns transcribed by the frequency spectrum generated by CWT scalogram.

**Figure 6:**
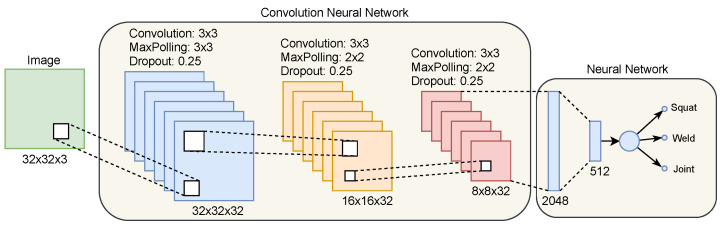
Architecture diagram of a convolutional neural network.

Figure 6 shows an illustration of the architecture of a CNN. It works similarly to a classical Multi-Layer Perceptron (MLP) network, where the layers contain models of neurons [23]. The initial layers have extracting features, being commonly called feature maps. Convolution is a linear process and allows the extraction of relevant information to the classification. The subsampling layer (pooling) spatially reduces the data size by maintaining the dominant characteristics that are invariant. The pooling layer reduces the computational effort, which is specially important for the training process. The final layer performs the classification. It uses the features extracted from the image as inputs to identify patterns. The definition of parameters during a CNN project follows: kernel dimension, how borders are processed; step size in convolution; and kernel quantity and type [21].

The CNN architecture implemented for RSDVS receives the scalogram generated through CWT as the input image. The images are resized to 32 × 32 pixels and separated into three layers of Red Green Blue (RGB) colors (an acronym for the additive color system combining Red, Green and Blue). The feature extraction stage occurs through two convolution sequences followed by subsampling. Thirty-two filters are used in the first convolution stage. After, the obtained characteristics feeds the inputs of the classification network. It generates a 512 length vector that serves as input for the classification step.

In Section 4, the data acquisition process is detailed, as well as the training and validation steps of the proposed method.

## 4. Method Validation

To validate the proposed method of detecting and identifying surface defects on the tracks, a series of field trials were carried out on the MRS Logística railway (company that has the railway concession in southeast region of Brazil). The device to attach the EC sensors to the proposed inspection vehicle in the RSDVS can be removed and used manually. EC acquisitions were performed manually to ensure enough statistic and to enable previously identification of the rail defects by experts. Note that the EC coupling with the rail was kept the same as in the vehicle, which contributes to maintain similar signal behaviour for both acquisitions.

**Figure 7:**
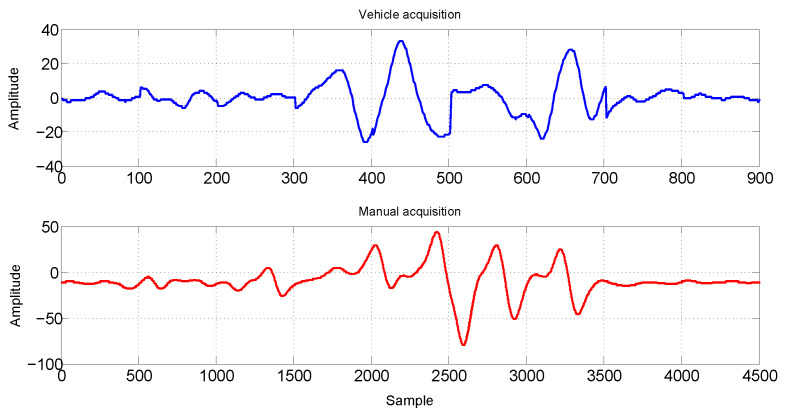
Comparison between manual (**top**) and vehicle acquisition (**bottom**). The signal behaviour is the similar for both acquisitions.

Figure 7 shows two examples of EC acquired signals with a sampling frequency of 7.5 kHz. The vehicle acquired signal can be seen on the bottom, and the one acquired manually can be seen on the top. Due to the higher velocity of the vehicle in comparison with the manual acquisition, the number of samples is 5 times smaller than the manual acquisition for approximately the same rail length, but the signal behaviour is similar for both acquisitions, showing that the manual acquisition can be used without loss of generality to design and validate the offline classification method. The data set used in this work is available in [24].

Table 1 shows the total number of samples acquired for each type of anomaly, which was used to design and validate the method. The data was split in two sets—training and validation—following the proportion of 75% for training and 25% for validation.

The samples were collected at different points of the railway. Small stretches were covered on the track, containing the evaluated defects. Therefore, it was possible to characterize visually the signals referring to the respective defects and those regions of the rails in normal conditions.

### 4.1. Defect Detection and Location

The embedded system for online detection and localization of rails defects based on EC signals were evaluated in field tests and will be detailed described in this section.

The first step of the signal defect detection and localization is the signal filtering, aiming to remove noise. Due to the probe coupling with the rail (see Figure 3), it is normal to have noise in the readings due to vibrations and electromagnetic interference.

**Figure 8:**
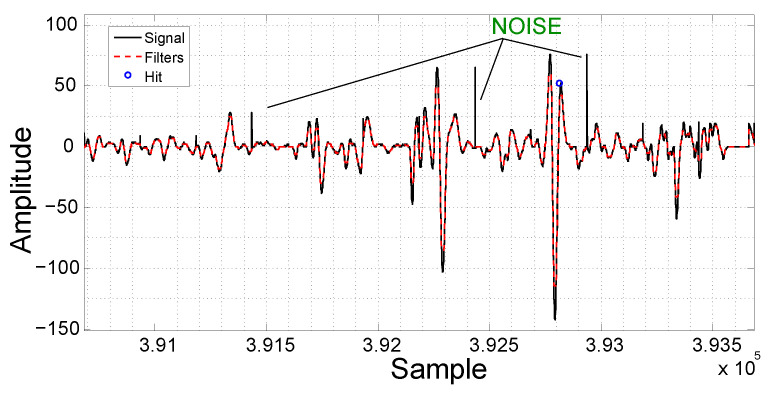
Example of spikes. The acquired signal can be seen in black and the filtered signal in red.

The noise called spikes is prevalent in the signal. A spike is an impulsive noise consisting of narrow peaks with relatively high amplitude [25] that can be caused by several sources of interference [26]. Figure 8 presents a sample of an acquired EC signal with spikes.

**Figure 9:**
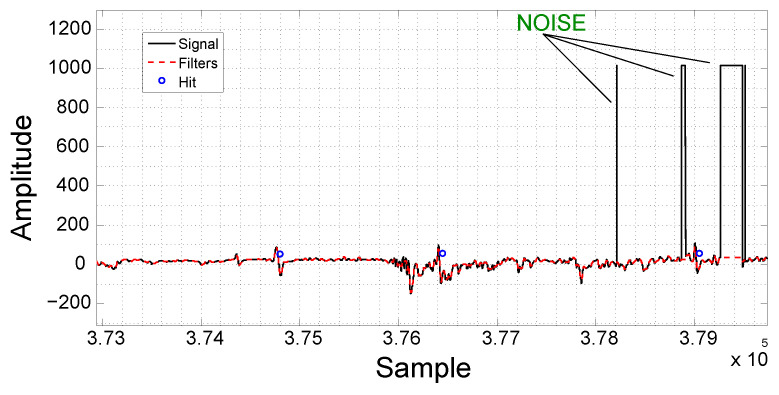
Example of Heaviside noise. The acquired signal can be seen in black and the filtered signal in red.

Another kind of noise that can be found in the acquired signal is similar to a high-amplitude step and can be seen in Figure 9. This kind of noise can be modeled by a Heaviside function [27] and consists of a sudden elevation in the signal level due to the EC sensor probe trepidation.

In order to remove the spikes from the acquired signal, a median filter was used [28]. The median filter is simple but very powerful. It scans the signal with a sliding window of a given size and its output is the median of the neighboring samples inside the window. In this work, the size of the filter window was determined empirically using the training data set. The selected window size was 21, which is enough to remove the spikes, preserving the remaining signal characteristics. Figure 8 shows an example of the application of the median filter in a sample of the signal corrupted by spikes. The acquired signal can be seen in black and the filtered signal in red.

**Figure 10:**
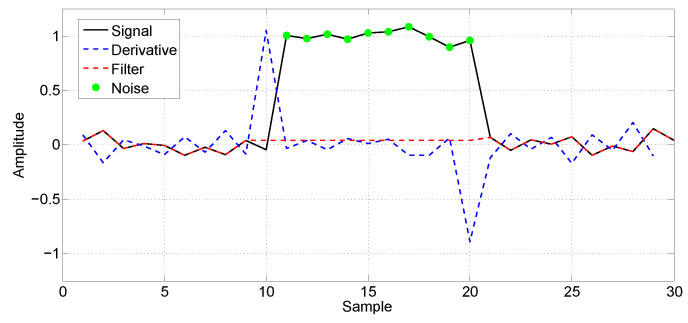
Heaviside noise filtering process. The acquired signal is depicted in black, the derivative in blue and the filtered signal in red.

After the removal of the spikes using the median filter, the signal is still corrupted by the Heaviside noise. Therefore, further processing is required. Due to the abrupt transitions of the Heaviside noise, it is possible to identify this region using the first derivative. The pair of peak points in the derivative allows its localization. Once the region with Heaviside noise is located, the samples are replaced by zero. Figure 10 illustrates the application of the technique, where the acquired signal is depicted in black, the derivative in blue and the filtered signal in red.

**Figure 11:**
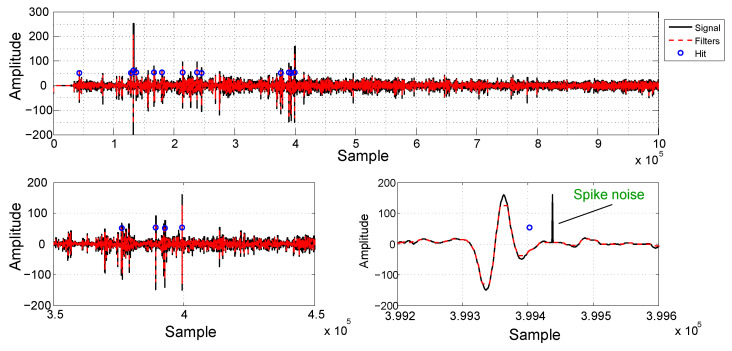
An example of the rail surface detection process. On the top, we can see a signal fragment of about 134 s long sampled at 7.5 kHz, where the horizontal axis indicates the sample number. The bottom plots are zooming versions of the signal. On the right plot, the blue circle indicates the location of the GPS stamp.

Figure 11 shows a signal fragment of ~134 s long (7.5 kHz sampling rate), envisaging to illustrate the online defect detection and localization process. In the top plot, we can see the acquired signal depicted in black, the filtered signal in red and blue circles indicating the detected defects. The horizontal axis indicates the sample number. The bottom plots are zooming versions of the signal, around sample 3994, in order to enhance visualization. On the right plot, the blue circle indicates the location of the GPS stamp. As already mentioned, the first step of the online detection is the signal offset removal, followed by the application of the median filter and the Heaviside filter. Finally, after the noise removal, the signal amplitude is compared with a pre-selected threshold. In this work, the threshold level was 50 counts, based on the training data set.

After the online defect detection, a window with a predetermined width is selected around the detection point. These signal windows are further processed by the CWT and then classified by the CNN. A window with 2000 samples was chosen based on the signal characteristics of the training data set.

### 4.2. Classification

The classification process begins with the window of the detected signals. The WPS is computed and used as input of the CNN. The WPS image is processed by the CNN as illustrated by the diagram shown in Figure 6. At the end of the CNN processing, the signal is classified as squat, weld or joint, the three classes considered in this work.

**Figure 12:**
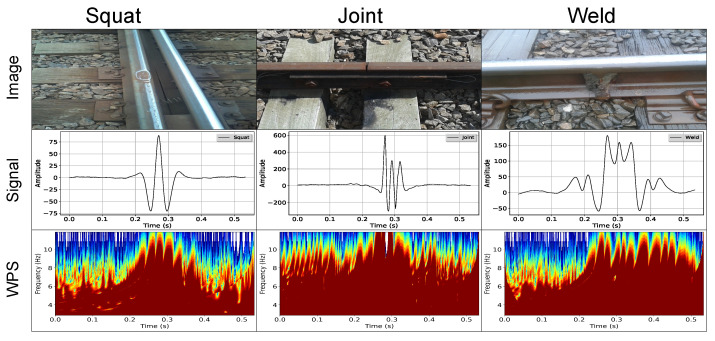
Characteristics of the anomalies considered in this work: squat (**left**), joint (**middle**) and weld (**right**). From top to bottom: picture of the anomaly, the EC acquired signal mean over the training data set and the WPS of the mean signal.

Figure 12 shows samples of the three classes divided into three columns. From top to bottom pictures of the anomalies, the mean of the EC acquired signal over the training data set and the WPS of the mean signal can be seen. Differences between the mean signal and WPS image for each type of anomaly can be noted, indicating that it is possible to identify the different classes based on the EC acquired signal, although this is not true for all events in the data set. Therefore, a machine learning approach for classification is justified.

**Figure 13:**
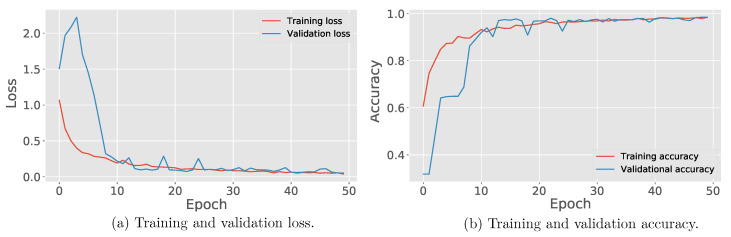
CNN loss (**a**) and accuracy (**b**) per epoch.

Figure 13 presents the learning curves for CNN training and validation. It shows the model’s performance in this process over time. In addition to the performance in the learning stage, curves allow assessing whether the separate data sets for training and validation are adequately structured [29]. In Figure 13a, the metric evaluated seeks to minimize losses or errors during the training and validation process. In this form of performance evaluation, learning is obtained by reaching the value near to zero on the vertical axis. Figure 13b shows that the learning process is evaluated by measuring accuracy in performing the classification. Therefore, reaching values near one indicates the effectiveness of the learning process. On both plots, it can be seen that the CNN learning curves were concluded around epoch 30.

**Figure 14:**
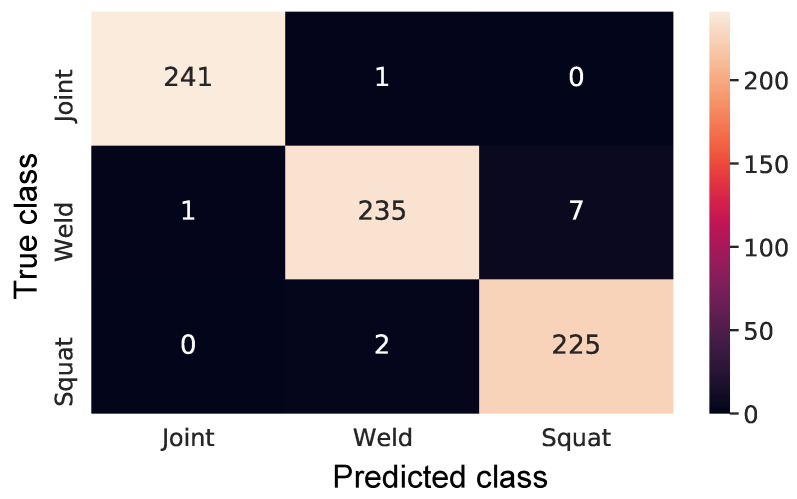
Classification confusion matrix.

The validation data set, as presented in Table 1, is processed by the trained CNN, and Figure 14 shows the confusion matrix for validation data set. The confusion matrix consists of a way to evaluate the performance of the classification process [30], where the vertical axis represents the label of a sample, while the horizontal axis the label predicted by the model. It can be seen only one error for joints, which was incorrectly assigned as weld; eight mistakes for welds, one assigned as a joint and seven as squats; and only two mistakes were found for squats, that were assigned as welds. The overall performance achieved was ~98%.

### 4.3. Comparison with Other Methods

A comparison with other classification methods commonly found on the literature is carried out in this section. The following methods were considered: Logistic Regression, Nearest Neighbors, Decision Tree, Extra Trees, Random Forest, MLP, Adaboost and Quadratic discriminant analysis (QDA).

**Figure 15:**
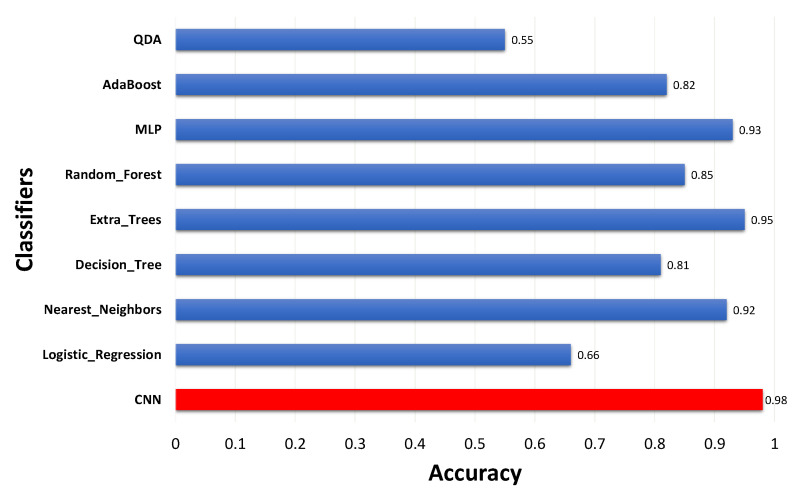
Classifiers’ accuracy comparison.

Figure 15 shows the classifiers’ overall performance. It can be seen that the proposed CNN presented the best result, with an accuracy of ~98%.

**Figure 16:**
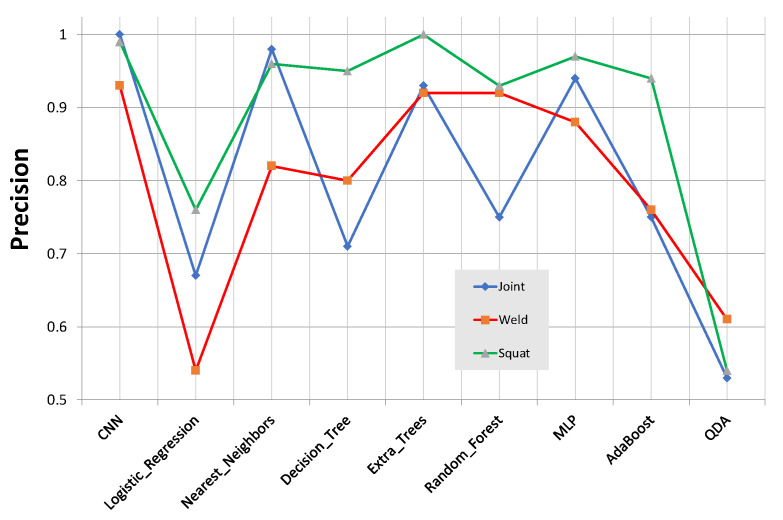
Classifiers’ precision comparison.

Figure 16 presents the classifiers’ precision. Again, the proposed CNN presented the highest precision among all classifiers. Therefore, we can expect better generalization for the CNN.

**Figure 17:**
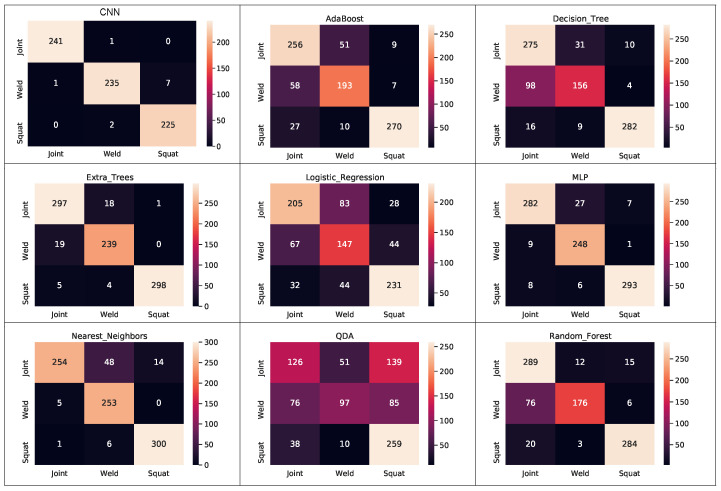
Classifiers’ confusion matrix.

Finally, the confusion matrix for each method can be seen in Figure 17.

Among the evaluated classifiers, the proposed CNN presented better overall performance and generalization capability.

## 5. Discussion

Note that the proposed embedded system and method were evaluated in field tests, going one step further to the works found on the literature, based on eddy current [6,12,31]. Moreover, the proposed CNN combined with WPS as preprocessing tool presented better accuracy and precision in comparison with other methods.

This solution applies to the studied problem, automatically solving defects detection proposed here and increasing the productivity of the field activity. As limitations, the method presents the need to train a CNN to classify the types of failure demands obtaining the respective characteristic signals of each type of failure evaluated. These signals are obtained from tests performed on the tracks. In regions with large extensions of tunnels, it can be challenging to obtain the geographical coordinates of the route and the points where surface defects are detected. This interference can be minimized by estimating coordinates by applying Kalman filters [32,33], and additionally including the Inertial Navigation System (INS) [34,35] combined with the GPS reading.

The probe is equipped with only one channel, where the respective sensor covers the entire surface of the track. More channels could better detail the profile, facilitating the identification of RCF.

Due to practical limitations related to the availability of the railway and service vehicles, it was very difficult to acquire large data sets and other types rail surface anomalies. Therefore, some further steps can still be done:

- improve the probe coupling with the rail, envisaging to improve flexibility;
- evaluate the proposed method for other types of rail surface anomalies; and
- estimate the rail surface anomaly intensity, based on the EC behaviour.

## 6. Conclusions

This work presented an embedded system to detect and identify surface rail defects. The mapping of the surface defects on the rails can aid the maintenance process and improve its performance, whereas the defect points and type are known. Thus, the grinding process of the rails acts only in the regions with defects, and the maintenance process can be carried out through the defects’ map which avoids unnecessary wear, increasing the useful life time of the rails. Moreover, the maintenance process can be carried out quickly, decreasing the railway line blocking time.

The proposed system can be easily embedded in any railway line inspection vehicles. The components are robust and easy to operate. The inspection routine performed by the system allows periodic evaluation with great agility.

The mapping of the inspection trajectory and the identified points are performed by a GPS, which helps in the maintenance process in the case of the squat defect and identifies points with joints and welds along the track. Additionally, the detection process captures images of the track during the inspection, which can be used to allow visual identification of defects or further processing.

The results from the field tests showed that the proposed method for rail defect classification, a CNN combined with WPS, showed better performance when compared with several other methods found on the literature.

Finally, the new embedded system assists the maintenance process of the railway tracks and allows the location and identification of surface defects on rails, adding speed to the grinding process, reducing losses and increasing the useful life of the rails. 

## Figures and Tables

**Table 1:** Data set separation.

Type	Total Samples	Total Validation
Squat	939	227
Joint	954	242
Weld	955	243
